# Development of cancer-associated fibroblast-related gene signature for predicting the survival and immunotherapy response in lung adenocarcinoma

**DOI:** 10.18632/aging.204774

**Published:** 2023-06-06

**Authors:** Yong Zhang, Fuyi Cheng, Jinhu Ma, Gang Shi, Hongxin Deng

**Affiliations:** 1 Department of Biotherapy, Cancer Center and State Key Laboratory of Biotherapy, West China Hospital, Sichuan University, Chengdu 610041, Sichuan, China

**Keywords:** lung adenocarcinoma, cancer-associated fibroblasts, immunotherapy, prognosis

## Abstract

The present study aims to construct a predictive model for prognosis and immunotherapy response in lung adenocarcinoma (LUAD). Transcriptome data were extracted from the Cancer Genome Atlas (TCGA), GSE41271, and IMvigor210. The weighted gene correlation network analysis was utilized to identify the hub modules related to immune/stromal cells. Then, univariate, LASSO, and multivariate Cox regression analyses were employed to develop a predictive signature based on genes of the hub module. Moreover, the association between the predictive signature and immunotherapy response was also investigated. As a result, seven genes (FGF10, SERINE2, LSAMP, STXBP5, PDE5A, GLI2, FRMD6) were screened to develop the cancer associated fibroblasts (CAFs)-related risk signature (CAFRS). LUAD patients with high-risk score underwent shortened Overall survival (OS). A strong correlation was found between CAFRS and immune infiltrations/functions. The gene set variation analysis showed that G2/M checkpoint, epithelial-mesenchymal transition, hypoxia, glycolysis, and PI3K-Akt-mTOR pathways were greatly enriched in the high-risk subgroup. Moreover, patients with higher risk score were less likely to respond to immunotherapy. A nomogram based on CAFRS and Stage presented a stronger predictive performance for OS than the single indicator. In conclusion, the CAFRS exhibited a potent predictive value for OS and immunotherapy response in LUAD.

## INTRODUCTION

Lung cancer is the most common malignancy and the leading cause of cancer-related deaths worldwide [1], posing a threat to human health. Lung adenocarcinoma (LUAD) is the main pathologic subtype of lung cancer. Nowadays, immunotherapy represented by anti-PD-1/PD-L1 has yielded a considerable clinical benefit for patients with various cancer types [2–5], including lung cancer. However, most patients showed minimal or no efficacy to immunotherapy, which is far from the clinical need [6]. It is necessary to screen patients sensitive or resistant to immunotherapy. An elevated PD-L1 expression, high tumor mutation burden (TMB), and microsatellite instability-high (MSI-H)/deficient mismatch repair (dMMR) are considered to be positive indicators for immunotherapy [7]. However, it is insufficient to screen suitable candidates for immunotherapy using these approved biomarkers. Therefore, it is urgently recommended to develop comprehensive indexes to predict the survival probability and clinical response to agents.

The tumor microenvironment (TME) has attracted increasing attention due to its crucial roles in angiogenesis, metastasis, and the therapeutic response [8–10]. The TME is mainly composed of immune cells (lymphocytes, dendritic cells, macrophages), stromal cancer-associated fibroblasts (CAFs), endothelial cells, extracellular matrix (ECM), and soluble signaling molecules [11–14]. As an essential component of TME, CAFs exhibit heterogeneity and versatility in function [8]. CAFs have been reported to elicit carcinogenesis and facilitate progression through multiple pathways. First, CAFs can facilitate tumor growth, angiogenesis, invasion, and metastasis by CAF-derived soluble molecules, and recruit suppressive immune cells by TGF-β and hepatocyte growth factor (HGF) [15]. Moreover, CAFs can degrade ECM by releasing matrix metalloproteinases (MMPs) and generating new matrix proteins, thus remodeling the TME in favor of immune evasion [16, 17].

Basic and translational research had verified the efficacy of a promising targeted therapy against CAFs [18, 19]. Pioneering studies have created a CAF-related gene risk signature (CAFRS) to predict the survival rate of gastrointestinal cancers [20, 21]. So far, a predictive model composed of CAF-related genes has not yet been constructed or validated in LUAD. Herein, we performed a weighted gene co-expression network analysis (WGCNA) to screen highly correlated gene modules and constructed a Cox regression model composed of CAFs-related genes to predict the prognosis and the immunotherapy response in LUAD. To gain a more powerful and reliable predictive model, we developed a novel prognostic nomogram that combined clinical features and risk score calculated by the CAFRS.

## MATERIALS AND METHODS

### Data acquisition and preprocessing

The transcriptome and somatic mutational profiles and clinical data of LUAD were extracted from TCGA-GDC (https://portal.gdc.cancer.gov/). GSE41271, with clinical information obtained from GEO, was treated as the external testing group. The immune-related functional gene sets were downloaded from GSEA (http://www.gsea-msigdb.org/gsea/index.jsp). The transcriptome profiling and clinical variables of the IMvigor210 cohort were curated from a freely available software and data package that can be downloaded from http://research-pub.gene.com/IMvigor210CoreBiologies. Raw count data were first transformed into the TPM value. Our study did not require approval from the ethics committee, as it used open-access data retrieved from the TCGA or GEO database.

### Abundance of tumor-infiltrating immune cells

The MCPcounter algorithm was utilized to quantify 11 immune/stromal cells (T cells, CD8^+^ T cells, cytotoxic lymphocytes, NK cells, B lymphocytes, monocytes, macrophage, myeloid dendritic cells, neutrophils, endothelial cells, and CAFs) [22, 23]. The fractions of intratumoral immune/stromal cells using distinct algorithms, including MCPcounter, were obtained from TIMER online (http://timer.cistrome.org/) [24]. In addition, the proportions of the 16 immune cell types were evaluated via the CIBERSORT algorithm [25].

### Selection of hub modules related to immune or stromal cells

The R package of WGCNA was employed to construct the weight co-expression network based on the gene expression matrix of TCGA-LUAD. Briefly, a similarity matrix containing the expression levels of paired genes was generated and then converted into the adjacency matrix based on the adjacency between the paired genes. Parameter β was then defined and used to construct a weighted proximity matrix that matched the gene distribution. We performed hierarchical clustering with the minimum size to build dynamic trees. Module eigengenes (MEs) and clustering heatmaps were used to characterize the hub modules. The correlation analysis between the module genes and fraction of immune/stromal cells was calculated using Pearson’s test [26, 27]. Through this, the hub gene modules related to the infiltrating immune/stromal cells were identified.

### Construction and validation of CAFRS

A total of 457 LUAD patients from TCGA were randomly partitioned into the training and testing cohorts using the R “caret” package, according to the 1:1 ratio. Then, the candidate genes related to the immune/stromal cells were incorporated into the univariate Cox regression to identify the survival-related genes, followed by a LASSO regression analysis to exclude the overfitting genes. Based on the screened survival-related genes, a multivariate Cox regression analysis using forward and backward regression analyses was employed to construct the predictive model in the training cohort. The risk score of each patient was calculated using the following formula: Σgene expression level × regression coefficient.

LUAD patients from TCGA training, TCGA testing, and GEO cohorts were divided into high- and low-risk groups using the median value of the TCGA training cohort as the cutoff. Kaplan–Meier survival curves between high- and low-risk subgroups and time-dependent receiver operating characteristic (ROC) curves in three independent cohorts were plotted using R packages of “survival,” “survminer,” and “timeROC”. Moreover, the risk score and survival status of each individual were shown by the risk curves, patients’ survival scatter plots, and heatmaps.

### Mutation landscape, gene set variation analysis (GSVA), and single sample gene set enrichment analysis (ssGSEA)

The R “maftools” package was used to analyze the mutation landscape of the top 15 genes and Epidermal growth factor receptor (EGFR) between the high- and low-risk subgroups. The survival difference of the subgroups stratified by the TP53 status and risk scores were computed. Referring to the hallmark gene set, the expression matrix of all genes was converted into the enrichment score of key oncogenic pathways/phenotypes using the R “gsva” package (method = “gsva”). Then, differentially expressed scores of the pathways/phenotypes were determined between the high- and low-risk subgroups using the R “limma” package. Similarly, the gene expression matrix was respectively converted into the proportions of immune cells and scores of immune functions using the R “gsva” package (method = “ssgsea”) and analyzed between the low- and high-risk subgroups using the Wilcox.test and presented as bar plots.

### Prediction of the clinical response to immunotherapy or chemotherapy

Charoentong et al. [28] created a scoring scheme based on the gene sets of immune effector/suppressor cells, immune checkpoints, and the major histocompatibility complex to quantify the clinical response to immunotherapy, which was termed the immunophenoscore (IPS). In our study, the IPS of each patient was calculated. Furthermore, the following independent dataset was analyzed in our study: advanced urothelial carcinoma treated with anti-PD-L1 (IMvigor210) [29]. The proportion of different immunotherapy responses, including the complete response (CR), partial response (PR), stable disease (SD), and progressive disease (PD), were compared between high- and low-risk subgroups. Meanwhile, Kaplan–Meier curves of the overall survival (OS) stratified by CAFRS and ROC curves were delineated to present the predictive performance in OS and response to immunotherapy.

To identify the potential compounds that benefit from CAFRS, we calculated the half maximal inhibitory concentration (IC50) of compounds obtained from the Genomics of Drug Sensitivity Cancer website (https://www.cancerrxgene.org/) in LUAD patients using the R “pRRophetic” package and compared the IC50 values of compounds in the high- and low-risk subgroups. A low IC50 indicated a sensitive drug response.

### Construction and validation of a nomogram for OS

Based on the independent prognostic variables, including risk signature and other clinical variables (*P* < 0.05), a novel nomogram was constructed for LUAD patients in the TCGA group using the R package “rms.” Calibration curves for the survival probability of 1, 3, and 5 years were plotted to ascertain the precision and accuracy of the synthesis model. The nomogram’s diagnostic capacity compared to other clinical variables was tested via the area under the curve (AUC) value of the ROC curves.

### Expression verification of hub genes

A total of eight paired LUAD tumor and adjacent nontumor specimens were collected from the lung cancer center in West China Hospital. All patients provided their informed consent, and this work was approved by the Institutional Research Ethics Committee of West China Hospital. A quantitative real-time PCR (qRT-PCR) was employed to detect the mRNA expression of four candidate genes (SERPINE2, FGF10, LSAMP, PDE5A). Briefly, the total RNA was extracted from paired lung cancer tissues using TRIzol reagent (Invitrogen, USA). cDNA was synthesized from the total RNA using reverse transcription kits (TaKaRa, Japan). qRT-PCR was performed according to the manufacturer’s protocol using the TB Green Premix Ex Taq (TaKaRa, Japan). The primer sequences were obtained from Primer Bank (https://pga.mgh.harvard.edu/primerbank). The forward primer sequences of SERPINE2, FGF10, LSAMP, and PDE5A were as follows: 5`-TGGTGATGAGATACGGCGTAA-3`, 5`-CATGTGCGGAGCTACAATCAC-3`, 5`-AGAGTTCAGCCGGATCGGAA-3`, and 5`-GCAGAGTCCTCGTGCAGATAA-3`. The corresponding reverse primer sequences were as follows: 5`-GTTA GCCACTGTCACAATGTCTTT-3`, 5`-CAGGATGCTGTACGGGCAG-3`, 5`-CGTGCCTCGGTTAAAATCCAC-3`, and 5`-GTCTAAGAGGCCGGTCAAATTC-3`. The product length of these primers was 101 bp, 138 bp, 105 bp, and 83 bp, respectively.

### Statistical analysis

All data were processed under R language (Version 3.6.1), and all statistical methods were conducted using the corresponding R packages. The Wilcox test was applied to compare the nonnormally distributed numerical variables, such as the gene expression level, proportion of immune/stromal cells, and scores of calculated pathways/phenotypes. When *P* < 0.05, the results were considered statistically different.

## RESULTS

### Construction of the weighted co-expression network and identification of the hub module

**Figure 1:**
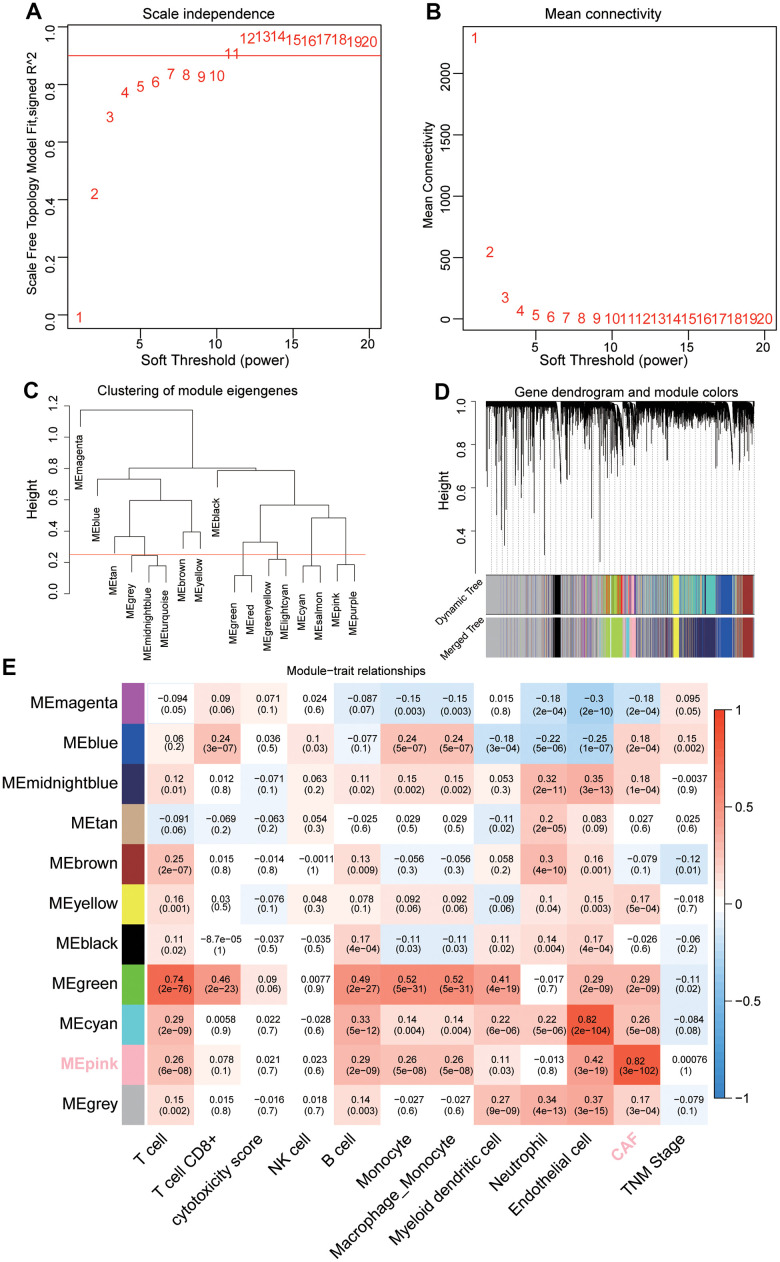
**Sample clustering and identification of CAFs-related module eigengenes (MEs) based on TCGA-LUAD.** (**A**, **B**) Analysis of the average connectivity of 1-20 soft threshold power. (**C**) Gene clustering dendrogram containing 17 MEs. (**D**) Merged gene clustering dendrogram containing 11 MEs. (**E**) A heatmap showing module-trait relationships. Each row and each column correspond to a module eigengene and immune/stromal cells, respectively.

Through data preprocessing, a total of 16, 306 genes with large fluctuation (*SD* > 0.8) and 425 out of 477 patients were selected for further analysis. The fraction of intratumoral immune/stromal cells were quantified using the MCPcounter algorithm. Using Pearson’s correlation coefficient, we performed a co-expression analysis between the above candidate genes and infiltration of the immune/stromal cells. The threshold soft power was calculated, and value 5 was regarded as the optimal value to construct the hierarchical clustering tree (Figure 1A, 1B). A total of 17 modules were merged into 11 modules based on the module-separating threshold of 0.25 (Figure 1C, 1D). Finally, we chose the pink module (closely related to CAFs) containing 580 genes for the subsequent predictive model construction due to the highest correlation coefficient (*R* = 0.82, *P* <0.001) with CAFs (Figure 1E).

### Determination of the CAFRS and validation of the predictive capability

**Figure 2:**
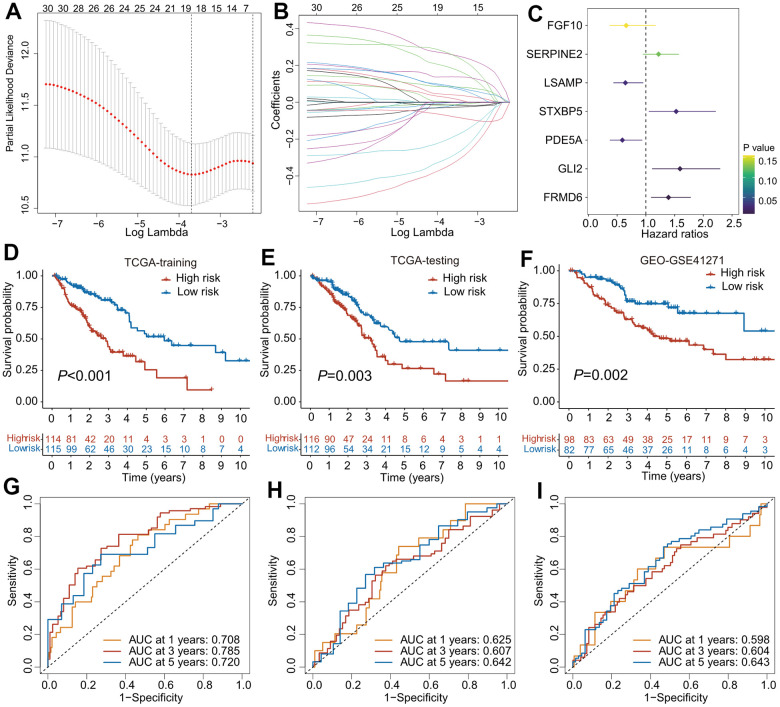
**The construction of predictive model for overall survival based on CAFs-related genes.** (**A**) Partial likelihood deviance of variables displayed by the Lasso regression model. The red dotted gray lines and two vertical lines represented the partial likelihood of deviance values, respectively. (**B**) Lasso coefficient profiles of 30 OS-related genes in TCGA training cohort. (**C**) Forest plot of CAFs-related risk signature (CAFRS) consisting of 7 genes identified by the multivariate Cox regression model. (**D**–**F**) Kaplan-Meier curves of patients from TCGA training, TCGA testing and GEO cohorts stratified by low- and high-risk subgroups. (**G**–**I**) Receiver operating characteristic (ROC) curves with 1-, 3-, and 5- year AUC values in the TCGA training, TCGA testing and GEO cohorts.

The TCGA cohorts were randomized into training (*n* = 229) and internal validation (*n* = 228) subgroups. Clinical features of these two cohorts and GEO-GSE41271 cohorts were listed in Table 1. The predictive model was first constructed in the training cohort. A total of 580 CAFs-related genes were first incorporated into the univariate Cox regression analysis, and 31 survival-related genes were determined. To avoid overfitting of the predictive model, the survival-related genes were assessed using a LASSO regression analysis (Figure 2A, 2B), and 19 genes were screened out for the subsequent multivariate Cox regression analysis. Finally, seven genes (FGF10, SERINE2, LSAMP, STXBP5, PDE5A, GLI2, FRMD6) were identified and used to establish a predictive model (Figure 2C). The risk scores of CAFRS were calculated as follows: risk score = -0.420 × FGF10 + 0.199 × SERINE2 + -0.441 × LSAMP + 0.423 × STXBP5 + -0.527 × PDE5A + 0.466 × GLI2 + 0.331 × FRMD6. In addition, Kaplan–Meier curves showed that patients with a high expression of SERINE2, STXBP5, GLI2, and FRMD6 had a poor prognosis, while those with a low expression of FGF10, LSAMP, and PDE5A had a favorable prognosis (Supplementary Figure 1A–1G). The ROC curves illustrated that the three-year AUC of CAFRS reached 0.694, which is greater than that of each gene (Supplementary Figure 1H).

**Table 1:** Patients’ characteristics of TCGA-training, TCGA-testing and GSE41271.

**Characteristics**	**TCGA-training**	**TCGA-testing**	**GSE41271**
**Number of cases**	229	228	180
**Age**	65.41±10.25	64.44±9.91	63.62±10.44
**Gender**			
Female/male	119/110	130/98	90/90
**TNM stage**			
I/II/III/IV	119/55/40/11	128/49/34/13	97/29/50/4
**T stage**			
T1/T2/T3/T4	83/119/17/9	72/125/20/8	NA
**Lymph node**			
N0/N1	146/78	149/73	NA
**Metastasis**			
M0/M1	167/11	140/12	176/4

To validate the accuracy and reliability of the predictive model, patients from three independent cohorts were divided into low- and high-risk subgroup using the median value as the cutoff. The Kaplan–Meier survival curves found that patients with high-risk scores had a poorer outcome than those with low-risk scores, regardless of whether the TCGA training, TCGA testing, or GEO cohort was used (Figure 2D–2F). The ROC analysis revealed that the AUC values of 1-, 3-, and 5-year OS in the TCGA training cohort were 0.708, 0.785, and 0.720, respectively (Figure 2G), while the AUC values were 0.625, 0.607, and 0.642 for the 1-, 3-, and 5-year OS, respectively, in the TCGA testing cohort (Figure 2H). In the GEO cohort, the AUC values of the 1-, 3-, and 5-year OS were 0.598, 0.604, and 0.643, respectively (Figure 2I). The distribution of the patients` risk scores, survival status, and expression heatmaps of the prognostic genes in these three cohorts are shown in Supplementary Figure 1I–1K.

**Figure 3:**
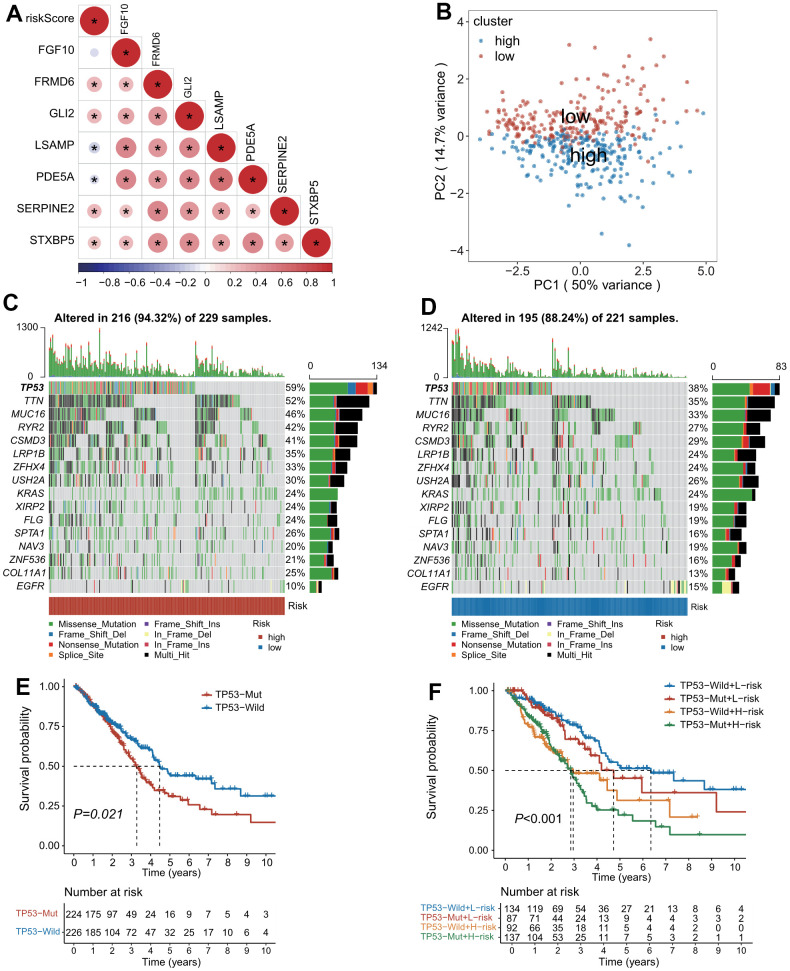
**The landscape of genetic alterations stratified by CAFs-related risk signature (CAFRS).** (**A**) A correlation diagram of risk score and expression levels of 7 CAFRS genes. (**B**) Principal component analysis of seven CAFRS genes to classify patients into low-risk and high-risk clusters. (**C**, **D**) Oncoplots depicting the top 15 mutational genes and EGFR between low- and high-risk subgroups. (**E**) Kaplan-Meier curves of all LUAD patients stratified by TP53 (with highest mutation rate) status. (**F**) Kaplan-Meier curves of all LUAD patients stratified by TP53 status and CAFRS scores.

In addition, the riskscore was positively correlated with the expressions of poor prognostic genes-FRMD6, GLI2, SERPINE2 and STXBP5, but negatively correlated with the expressions of favorable prognostic genes-LSAMP and PDEA5 (Figure 3A). The expression profiling of the seven genes composed of the predictive model demonstrated distinct clustering which corresponds to low- and high-risk subgroup (Figure 3B).

### Mutation landscape associated with CAFRS

The top 15 genes with high mutation rates (TP53, TTN, MUC16, RYR2, CSMD3, LRP1B, ZFHX4, USH2A, KRAS, XIRP2, FLG, SPTA1, NAV3, ZNF536, COL11A1) combined with EGFR were displayed as waterfall plots in both the high- and low-risk subgroups. Patients with mutations of these genes accounted for 94.32% and 88.24% in the high- and low-risk subgroups, respectively. Of note, the mutation rate of TP53 was 59% and 38% in the high- and low-risk subgroups, respectively, whereas the mutation rate of EGFR was 10% and 15% in the high- and low-risk subgroups, respectively (Figure 3C, 3D). As illustrated by the Kaplan–Meier curves, patients with a TP53 mutation had a shorter OS than those with the TP53 wild type (Figure 3E). Moreover, a combination of the risk score and TP53 mutation status could improve the predictive performance for OS because patients with the TP53 mutation type and high-risk scores had the worst outcome, while patients with the TP53 wild type and low-risk scores had a survival advantage (Figure 3F).

### Clinical characteristics associated with CAFRS

**Figure 4:**
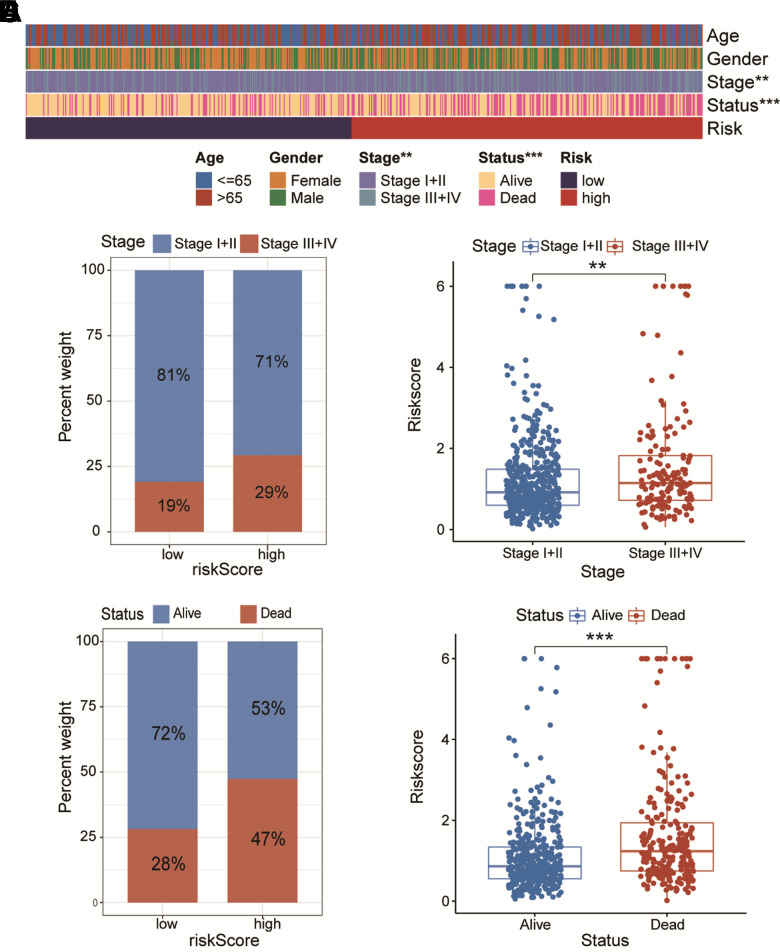
**The association of CAFs-related risk signature (CAFRS) with clinicopathologic features.** (**A**) The heatmap illustrating the correlation between CAFRS and Age, Gender, TNM stage and survival status. (**B**) The fraction of patients with distinct TNM stage in low- and high-risk subgroup. (**C**) The riskscores between stage I+II and stage III+IV subgroups. (**D**) The fraction of patients with distinct survival status in low- and high-risk subgroup. (**E**) The riskscores between alive and dead patients. (^**^P < 0.01, ^***^P < 0.001).

As illustrated in Figure 4A, the high- and low-risk clustering was remarkably associated with the pathologic TNM stage and survival status. In particular, the proportion of patients with stage III+IV was higher. in the high-risk group than that in the low-risk group, while the reverse was true for patients with stage I+II Patients with stage III+IV had a remarkably higher riskscore than those with stage I+II (*P*<0.01, Figure 4B, 4C). Similarly, the proportion of patients dead was higher in the high-risk group than that in the low-risk group, while the reverse was true for patients alive. Patients dead had a remarkably higher riskscore than those alive (*P*<0.001, Figure 4D, 4E).

### Phenotype characteristics associated with CAFRS

**Figure 5:**
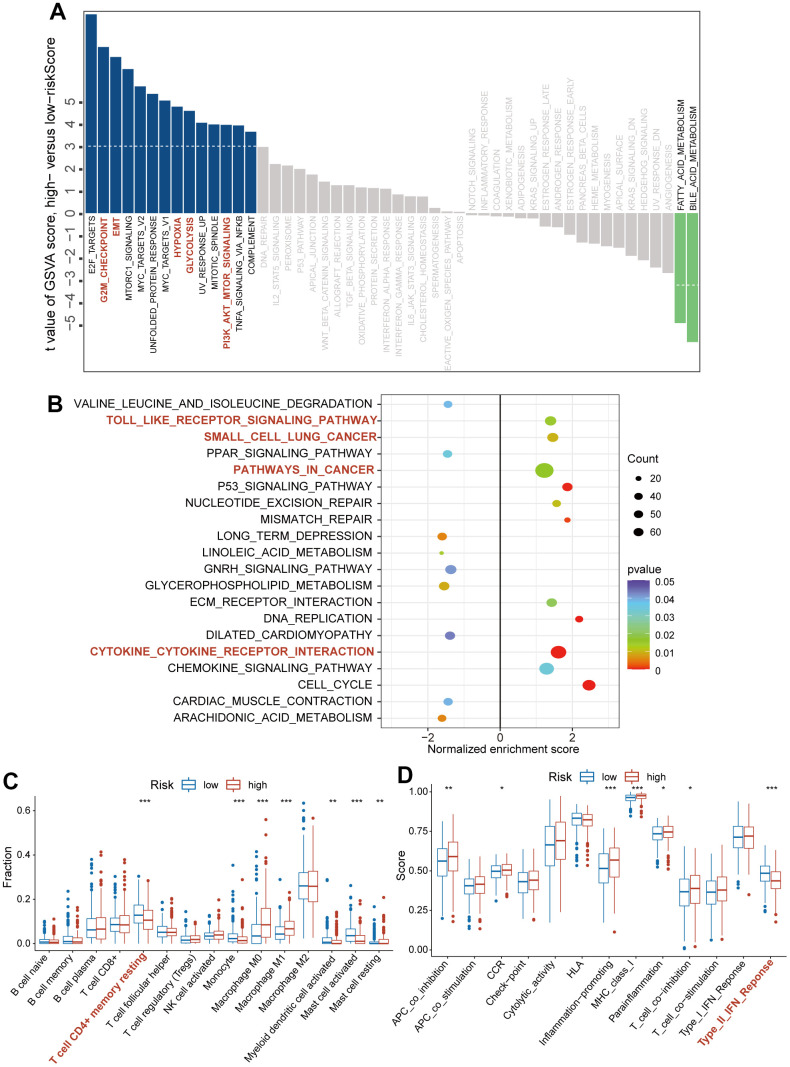
**The association of CAFs-related risk signature (CAFRS) with oncogenic pathways and immune cells/functions.** (**A**) The bar plots showing the GSVA scores of hallmark pathways (Fifty items) between the low- and high-risk subgroups. Red bar plots represent activated pathways in the high-risk subgroup. (**B**) The bubble plot indicating the activated and repressed pathways between low- and high-risk subgroup, referring to the KEGG gene set. (**C**) The fraction of tumor infiltrating immune cells between low- and high-risk subgroups (**D**) Immune function scores between low- and high-risk subgroup. (^*^P < 0.05, ^**^P < 0.01, ^***^P < 0.001).

To explore the underlying mechanism by which CAFRS affects carcinogenesis and progression of LUAD, we analyzed the association of the risk score with the pathways/phenotypes. Through a differential GSVA score analysis between the low- and high-risk subgroups, 16 of 50 hallmark gene sets were observed to be remarkably altered (*P* < 0.01). In particular, the G2M checkpoint, epithelial-mesenchymal transition (EMT), hypoxia, glycolysis, PI3K-Akt signaling pathways were greatly enriched in the high-risk subgroup, while the fatty acid and bile acid metabolisms were enriched in the low-risk subgroup (Figure 5A). In addition, “Toll like receptor signaling pathway”, “Small cell lung cancer”, “pathways in cancer” and “Cytokine-cytokine receptor interaction” were significantly activated in the high-risk group when Kyoto Encyclopedia of Genes and Genomes (KEGG) was taken as the reference gene set (Figure 5B).

We quantified the proportion of intratumoral immune cells using the CIBERSORT algorithm and scored the immune functions using the ssGSEA method. Based on the matrix of immune cells and immune functions, the differential analysis between the high- and low-risk subgroups was performed using the Wilcox.test, and the results were presented as bar plots. As shown in Figure 5C, 5D, the high-risk subgroup was characterized by decreased infiltration of the CD4^+^ T cell memory resting and enrichment score of type II IFN response (*P* < 0.001), which indicated an immunosuppressive TME.

### The role of CAFRS in predicting the benefits of immunotherapy and chemotherapy

**Figure 6:**
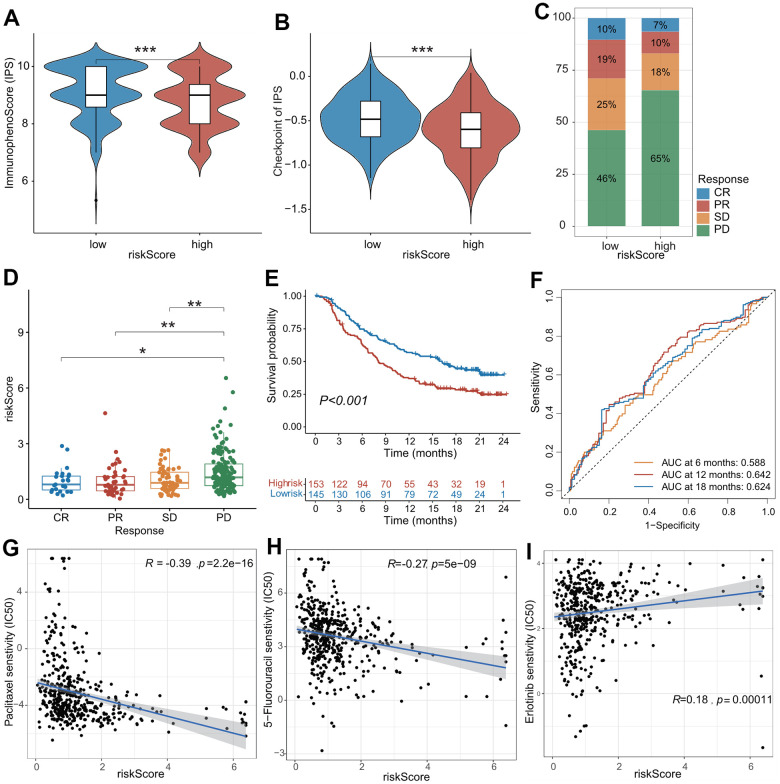
**The role of CAFs-related risk signature (CAFRS) in predicting drug response.** (**A**, **B**) The immunophenotype score (IPS) and one of its constituents-immune checkpoint score between low- and high-risk subgroups from TCGA cohort. (**C**) The proportion of patients with clinical response to anti-PD-1 immunotherapy in low- and high-risk subgroups from IMvigor210 cohort. SD, stable disease; PD, progressive disease; CR, complete response; PR, partial response. (**D**) The riskscore of CAFRS in the CR, PR, SD and PD subgroups. (**E**) Kaplan-Meier curves of patients from IMvigor210 cohort stratified by low- and high-risk subgroups. (**F**) The ROC curves illustrating the AUC values at 6-, 12- and 18-month. (**G**–**I**) The plots illustrating correlations of IC50 values for Paclitaxel, 5-FU and Erlotinib with CAFRS scores in TCGA cohort. (^*^P < 0.05, ^**^P < 0.01, ^***^P < 0.001).

Immunotherapy, represented by anti-PD-L1/anti-CTLA-4, has caused a breakthrough in antitumor treatment. In addition to PD-L1, TMB, and MSI-H/dMMR, the IPS was strongly recommended to predict the response to immunotherapy. Herein, the IPS was significantly higher in the low-risk subgroup compared with the high-risk subgroup (*P* < 0.001), indicating that patients in the low-risk subgroup were more susceptible to immunotherapy (Figure 6A). Furthermore, patients in the IMvigor210 cohort were divided into high- and low-risk subgroups using the median value of the CAFRS risk score. Patients in the low-risk subgroup exhibited a marked clinical response advantage and prolonged survival rate. The proportions of CR, PR, SD, and PD were 10%, 19%, 25%, and 46% in the low-risk subgroup, respectively, compared with the proportions of 7%, 10%, 18%, and 65% in the high-risk subgroup (Figure 6C). Similarly, the risk score of patients with CR was significantly lower than that of patients with PR, SD, and PD (Figure 6D). When the clinical response was dichotomized into response (CR + PR) and non-response (SD + PD) groups, the ratio of response to non-response was 29/71 in the low-risk subgroup. However, it fell to 17/83 in the high-risk subgroup. The risk score of the non-response group was significantly higher than that of the response group (Supplementary Figure 2A, 2B). In addition, the Kaplan–Meier curves revealed that patients in the low-risk subgroup survived longer than those in the high-risk subgroup (Figure 6E), which is in agreement with the results obtained from three independent cohorts. The AUC values of the ROC curves at 6 months, 12 months, and 18 months were 0.588, 0.642, and 0.624, respectively (Figure 6F), suggesting that this CAFRS can predict the prognosis of LUAD. Moreover, the established risk score is a poor prognostic factor for OS, independent of TMB, TNM stage, sex, chemotherapy, and immune subtype in the IMvigor210 cohort (Supplementary Figure 2C).

We investigated the differences between the IC50 values for common drugs between the low- and high-risk subgroups. As shown in Figure 6G–6I, the CAFRS score was markedly negatively associated with the IC50 values for paclitaxel and 5-fluorouracil (common chemotherapeutic agents) and positively associated with the IC50 values for erlotinib (common EGFR tyrosine kinase inhibitor [TKI]). Moreover, the low-risk subgroup presented remarkably higher IC50 values for paclitaxel and 5-fluorouraci but had lower IC50 values for erlotinib (Supplementary Figure 2D–2F). This indicated that LUAD patients with low-risk scores were more sensitive to erlotinib therapy but benefited little from conventional chemotherapy, which is likely due to drug resistance. In summary, CAFRS could predict anticancer drug responses in patients with LUAD.

### Nomogram construction combined with clinical characteristics and CAFRS

**Figure 7:**
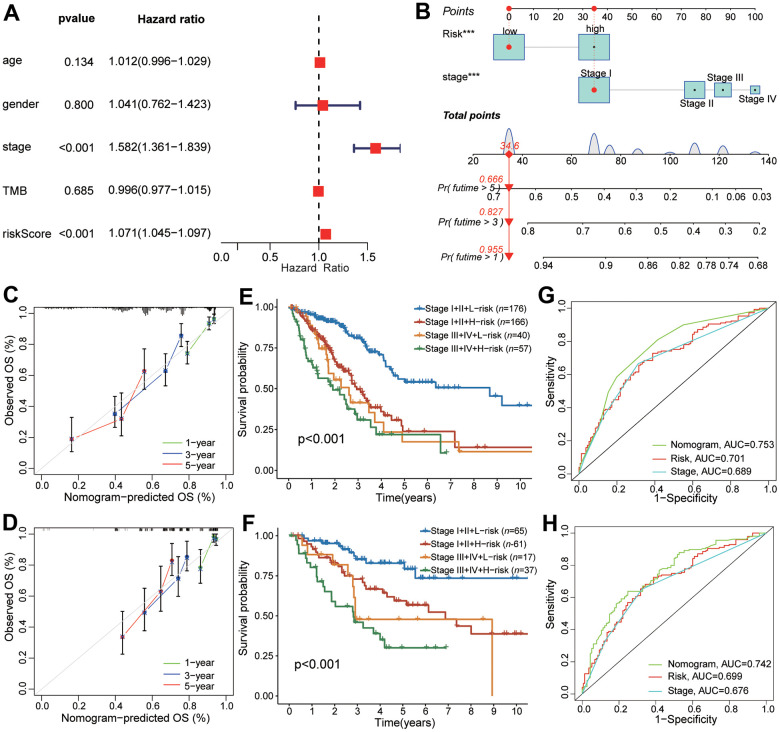
**The predictive performance of CAFRS in combination with TNM stage in OS for LUAD patients.** (**A**) The forest plot of multivariate Cox regression analysis for OS of TCGA-LUAD. (**B**) Nomogram of predicting 1-, 3-, and 5- year OS for TCGA-LUAD. (**C**, **D**) Calibration curves of the nomogram at 1-, 3-, and 5-year for LUAD patients from TCGA and GSE41271. Gray line indicates the ideal curve. The green, blue and red lines indicate bias-corrected curve at 1-, 3-, and 5-year. Dots are quartiles of our data set. (**E**, **F**) Kaplan-Meier curves of LUAD patients from TCGA and GSE41271 stratified by TNM stage and riskscore. (**G**, **H**) The ROC curves of TCGA and GSE41271 illustrating the AUC values of nomogram, CAFRS and TNM stage at 5-year.

The univariate and multivariable Cox regression analyses indicated that the CAFRS and TNM stages were independent prognostic factors for LUAD (Figure 7A). To explore a more accurate and reliable prediction tool, we established a nomogram to predict the survival probability of 1-, 3- and 5-year OS for LUAD (Figure 7B). During the 1-, 3- and 5-year OS, the calibration curves of the nomogram exhibited excellent concordance with the actual survival rate in two independent cohorts (TCGA-LUAD, GSE41271) (Figure 7C, 7D). The 5-year survival rate of stages I+II and the low-risk subgroup in the TCGA and GEO cohorts was up to 58.8% and 82.9%. However, the number of stages III+IV and the high-risk subgroup decreased, reaching 30% and 21.3%, respectively (Figure 7E, 7F). Moreover, compared with the single TNM stage or CRAFS, the merged nomogram had a better predictive performance in OS. The 5-year AUC value of the nomogram reached 0.742 and 0.702 in the TCGA and GEO cohorts, respectively, which is greater than that of the TNM stage and CAFRS (Figure 7G, 7H).

### mRNA verification of differential expression of CAFRS genes

**Figure 8:**
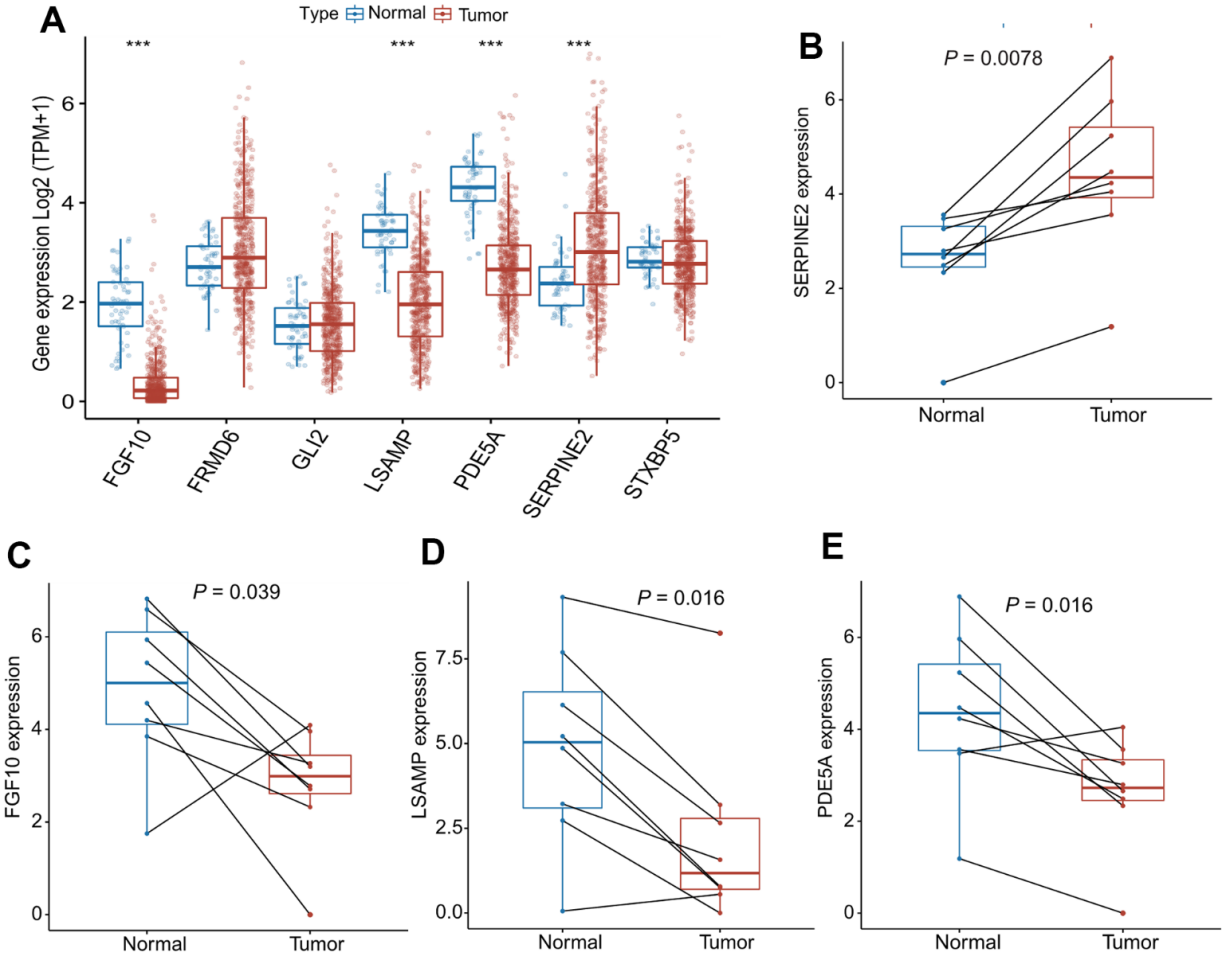
**mRNA expression of SERPINE2, FGF10, LSAMP, PDE5A in paired lung cancer tissues.** (**A**) The bar plots showing expression levels of seven genes composing the CAFRS from TCGA- LUAD. The asterisks represent the statistical *P* values (^***^P < 0.001). (**B**–**E**) Paired dot plots show four differentially expressed genes composing the CAFRS, further detected by qRT-PCR in paired lung cancer tissues. (^***^P < 0.001).

As shown in Figure 8A, four out of seven genes in the CAFRS were differentially expressed between the cancer and paratumor tissues from LUAD patients in TCGA. To prove the reliability of the CAFRS, the mRNA levels of these four genes were detected using a qRT-PCR. Consistent with the transcriptome data, SERPINE2 was more highly expressed in lung cancer tissues compared with that in paratumor tissues, while a low expression of FGF10, LSAMP, and PDE5A was found in lung cancer tissue (Figure 8B–8E).

## DISCUSSION

Over the past two decades, increasing evidence has indicated that cancer is not just a disease of altered genes but also a crosstalk between tumor cells and their TME, suggesting that TME plays a key role in carcinogenesis and progression [30]. Targeting TME could assist traditional therapies and improve clinical responses to agents in numerous cancer types [31]. However, the prevailing components of TME and proportions of immune or stromal cells vary among different cancer types. Herein, based on the bulk RNA-seq from TCGA, we identified 11 hub modules using the WGCNA method, analyzed their correlations with intratumoral immune/stromal cells, and proved CAFs to be the principal components of TME in LUAD. Similarly, a recent single-cell RNA-seq research has shown that CAFs are a major constituent with diverse molecular properties in breast [32], pancreatic [33], and lung cancer [34]. Moreover, CAFs derived from lung cancer are functionally heterogeneous, and classifications based on CAFs could predict an individual`s response to targeted therapy [35].

In the present study, we constructed a potent predictive model for prognosis and immunotherapy response using only seven genes, among which SERPINE2, STXBP5, GLI2, and FRMD6 were shown to be adverse prognostic factors for OS, while FGF10, LSAMP, and PDE5A were favorable prognostic factors. Some of these genes have been reported to exhibit not only tumor-promoting or tumor-inhibiting activities but also predictive indicators for survival or treatment outcomes in multiple cancer types. For example, serine proteinase inhibitor clade E member 2 (SERPINE2, also known as plasminogen activator inhibitor type 1) acted as oncogenes and promoted the proliferation, metastasis, or stemness behavior in various types of cancers [36–38]. As a biomarker of immunosuppression and fibrosis, SERPINE2 could serve as a poor prognostic factor for pancreatic cancer. Blocking the SERPINE2-related signaling pathway could overcome the resistance of chemo- or immunotherapy against pancreatic cancer [39]. Consistent with our study, increased SERPINE2 expression was correlated with a dismal prognosis of LUAD, and a high serum SERPINE2 concentration predicted a poor response to radiotherapy [40]. Zinc finger transcription factor GLI2 functions as the primary activator of the hedgehog signaling pathway, which is closely correlated with embryonic development and regeneration. However, the postnatal activation of the hedgehog pathway is characterized as contributing to tumorigenesis and progression of multiple cancer types [41–43]. Additionally, GLI2 induced chemoresistance in colorectal cancer through HIF-1α and TGF-β2 signaling pathways [44]. LSAMP inhibited cell migration via EMT and lower expression of indicated poorer prognosis for lung cancer [45]. PDE5A also belongs to a protective factor for the prognosis of colon cancer [46, 47]. The protumor role or anti-tumor role of FRMD6 varies among different cancer types. In lung cancer, FRMD6 promoted the tumor growth and invasion via mTOR pathway, while FRMD6, as a tumor suppressor inhibited the carcinogenesis and progression of prostate cancer and glioma [48–50]. Here, FRMD6 is a poor independent prognostic factor for lung cancer, in favor of the protumor role in lung cancer. Taken together, the protumor/anti-tumor roles of these predictive genes were based on increased/decreased property in expression or adverse/favorable property in prognosis. The exact role and mechanism underlying the lung carcinogenesis and progression remains unclear and further in-depth research is required.

Genetic mutations are considered initiating factors in tumorigenesis. To some extent, TMB reflects the prognosis and treatment outcome of cancer patients, especially for immunotherapy [51, 52]. Here, we investigated the relationship between CAFRS score and mutation frequency. and found TP53 mutation occurred more frequently in patients with high risk score, s, in agreement with a previous study that suggested TP53 mutation a poor prognostic factor [53]. Moreover, mutant TP53, but not wild TP53, binds to TANK binding protein kinase 1 (TBK1) and prevents the formation of TBK1-STING-IRF3 complex, which is required for the phosphorylation of IRF3 [54]. Phosphorylated IRF-3 then translocates to the nucleus and initiates the expression of type I IFN, which reflects the innate antitumor immunity. In summary, TP53 mutation contributed to immune evasion and patients with TP53 mutation are considered unsuitable for immunotherapy strategies [55].

Here, ssGSEA results indicated that the low-risk group was characterized by an increased number of T cells CD4^+^ memory resting, which has been reported to facilitate the initiation of antitumor immunity [56]. Patients with low risk score were more likely to undergo activation of type II IFN response, which reflects the antitumor immunity. In terms of the immunotherapeutic effect, the low-risk group had a higher IPS, indicating that these patients are more likely to benefit from immunotherapy. Moreover, IMvigor210, including 298 patients with urothelial cancer, was designed to investigate whether the PD-L1 expression affects the efficacy of immunotherapy. In our study, the proportion of CR and/or PR in the low-risk group was significantly higher than that in the high-risk group, indicating that patients with low risk score are more likely to benefit from immunotherapy. Similarly, Zheng et al. [57] constructed a four-gene CAFRS that could accurately predict the prognosis and therapeutic response to chemical agents and immune checkpoint inhibitors (ICIs) in gastric cancer. Patients with high-risk score were less likely to benefit from ICIs therapy in gastric cancer, but the result was not verified in an independent cohort with immunotherapy data. In addition, enrichment analyses proved that multiple oncogenic pathways/phenotypes such as PI3K/AKT, glycolysis, hypoxia and EMT were significantly activated in patients with high risk score. Taken together, enrichment analyses revealed the mechanism by which CAFRS score affect the prognosis and immunotherapy response.

Previous studies have shown that the combined strategy with clinical features could greatly enhance the accuracy and robustness in predicting the survival chance and treatment outcome [58, 59]. Here, we drew a nomogram by incorporating the CAFRS score and TNM stage which were both independent prognostic factors. The nomogram is a practical and portable tool for calculating the survival probability at each time point (e. g 1-year, 3-year, 5-year). Each continuous or categorial variable corresponds to its coefficient in the scoring scheme, we simply add the score value of every variable to get the combined score of a patient. A higher nomogram score indicates worse prognosis. The ROC curves indicated that the nomogram tool is more reliable than the single CAFRS score and TNM stage.

In conclusion, due to the crucial role of CAFs in lung cancer carcinogenesis and progression, we developed a risk signature CAFRS based on 580 CAF-related genes. The constructed CAFRS could predict both the survival chance and clinical response to chemo- and immunotherapy.

## Supplementary Material

Supplementary Figures
