# Supplementary material for: Development of cancer-associated fibroblast-related gene signature for predicting the survival and immunotherapy response in lung adenocarcinoma
Source: Aging (Albany NY). 2023 Jun 6;15(11):4986–5006. doi: 10.18632/aging.204774 (PMC10292873; doi:10.18632/aging.204774)
Supplement: Supplementary Figures [file aging-15-204774-s001.pdf]

SUPPLEMENTARY FIGURES

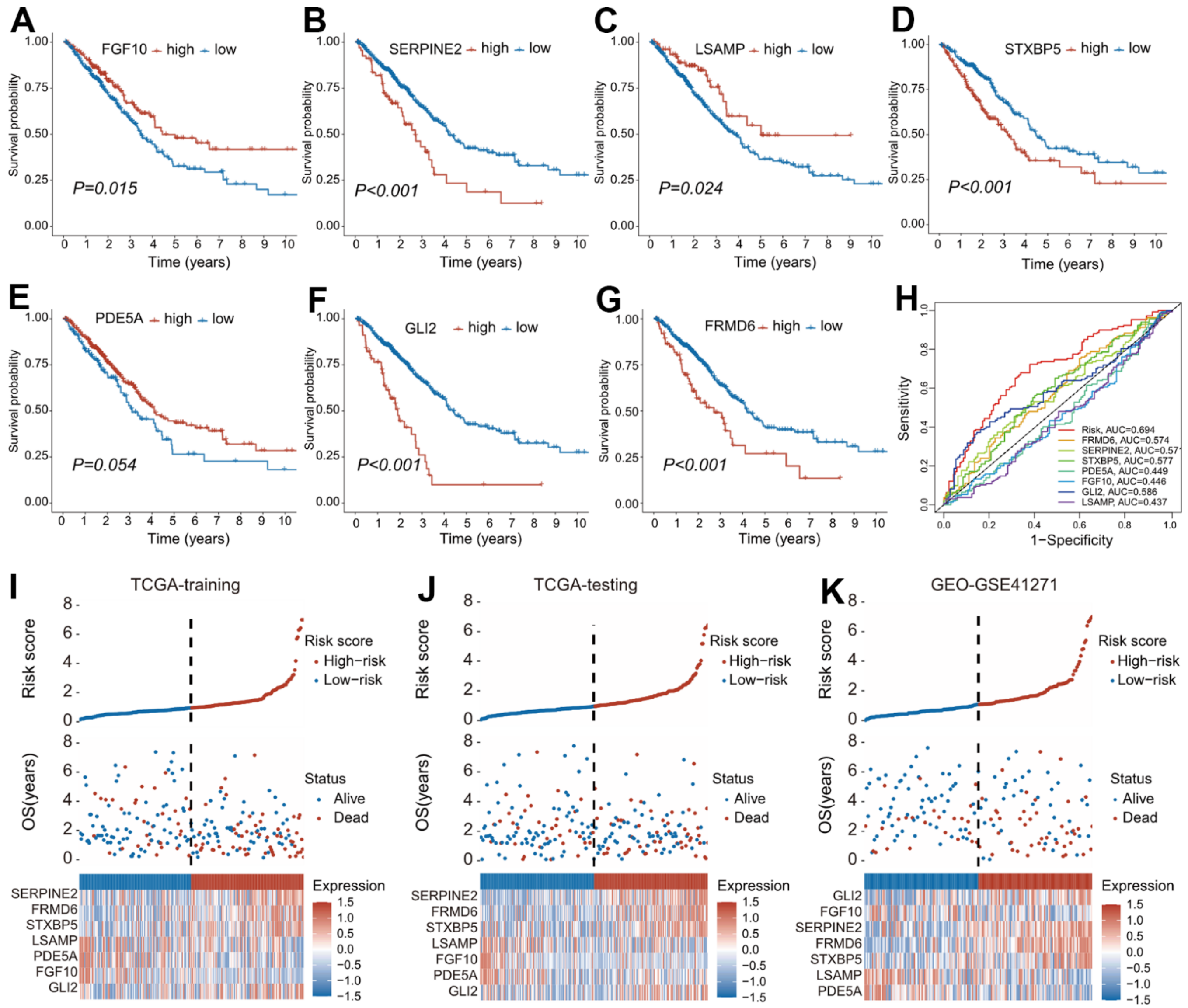

Supplementary Figure 1. The predictive performance of the model and seven composing genes. (**A**–**G**) Kaplan-Meier curves of patients stratified by expression profiling of 7 genes composing the CAFRS (**H**) Receiver operating characteristic (ROC) curves illustrating AUC values of 7 genes composing CAFRS at 3-year. (**I**–**K**) Distributions of survival status, risk score and expression profiles among patients from TCGA training, TCGA testing and GEO cohorts.

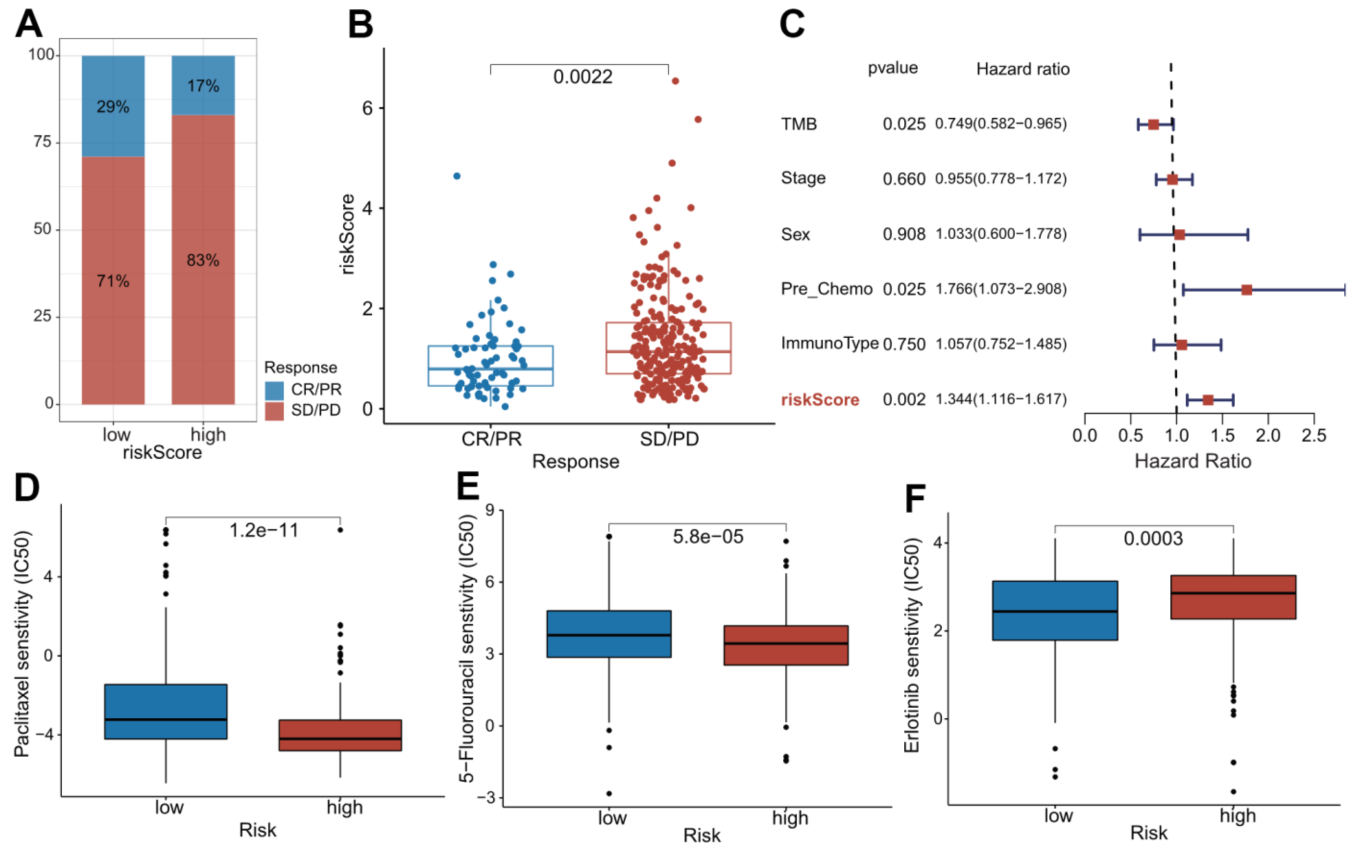

Supplementary Figure 2. The role of CAFRS in predicting the therapeutic response to drug therapy. (**A**) The proportion of patients with clinical response to anti-PD-1 immunotherapy in low- and high-risk subgroup from IMvigor210 cohort. SD, stable disease; PD, progressive disease; CR, complete response; PR, partial response. (**B**) Riskscores calculated by CAFRS in the CR+PR and SD+PD subgroups. (**C**) The forest plot of multivariate Cox regression for combined riskscore and clinical variables in IMvigor210 cohort. (**D**–**F**) Variations of responses to Paclitaxel, Erlotinib, 5-FU between low- and high-risk subgroups, respectively.
